# From MAX to MXene: Unveiling Robust Magnetism and Half-Metallicity in Cr_2_ZnC and Its Half-Metallic 2D Cr_2_C Through Ab-Initio Investigation

**DOI:** 10.3390/nano16020110

**Published:** 2026-01-14

**Authors:** Ahmed Lokbaichi, Ahmed Gueddouh, Djelloul Gueribiz, Mourad Rougab, Brahim Lagoun, Fatima Elhamra, Ahmed Mahammedi, Brahim Marfoua

**Affiliations:** 1 Laboratoire de Génie Des Procédés, Université Amar Télidji de Laghouat, Laghouat 03000, Algeria; 2 Department of Matter Sciences, Faculty of Science, Laghouat University, Laghouat 03000, Algeria; 3 Laboratoire de Physique des Matériaux, Université Amar Telidji de Laghouat, Laghouat 03000, Algeria; 4 Department of Science and Technology-Common Core, University Amar Telidji of Laghouat, Laghouat 03000, Algeria; 5 Department of Physics, Chemistry and Biology, Linköping University, SE-581 83 Linköping, Sweden

**Keywords:** MAX phase carbides, thermodynamic, dynamic, and mechanical stabilities, thermal properties, MXene nanosheets exfoliation, ab-initio method

## Abstract

A first-principles investigation was conducted to characterize the novel Cr_2_ZnC MAX phase and its exfoliated MXene nanosheet, Cr_2_C. The study critically examines the effect of electron correlations on the bulk phase, revealing that the PBE+U framework, unlike standard PBE, yields a dramatically enhanced magnetic moment of 12.80 μ_B_ (vs. 1.88 μ_B_), confirming the necessity of this approach for Cr-based carbides. The phase stability is confirmed through rigorous analysis of its thermodynamic, dynamic, and mechanical properties. For the derived 2D Cr_2_C, results confirm a robust half-metallic state with a total magnetic moment of 8.00 μ_B_, characterized by a metallic spin-majority channel and a semiconducting spin-minority channel with a 2.41 eV direct gap, leading to near-ideal spin polarization. These combined features establish Cr_2_C as a highly promising candidate for next-generation spintronic applications and 2D magnetic devices requiring room-temperature stability.

## 1. Introduction

MAX phases are layered, nanolaminated ternary carbides and nitrides with the general formula M_n+1_A X_n_ (commonly written M_n+1_AX_n_), where M = early transition metal (Ti, V, Cr, …), A = A-group (III-A/IV-A) element (Al, Si, Ga, …), and X = C or N (or mixtures). Typical values of n are 1 (211), 2 (312), and 3 (413). They combine ceramic-like (M-X) and metallic-like (M-A) bonding, giving a unique mix of properties (machinability, high stiffness and thermal stability, and good electrical/thermal conductivity) [1]. MAX phases crystallize in a hexagonal lattice (typical space group P6_3_/mmc). The structure can be viewed as stacks of edge-sharing M_6_X octahedra separated by A-element layers (the A layers are metallic and sit between the M-X blocks). The different n values change stacking thickness (M_2_AX = 211, M_3_AX_2_ = 312, M_4_AX_3_ = 413) [2]. The structure consists of rigid M-X octahedra and M-A triangular prisms, where the M-X bonds are shorter and stronger, with mixed covalent–ionic character, while the M-A interactions are longer and predominantly metallic. This dual motif explains many of the mixed properties [3]. The A and X sites tolerate substitution and vacancies; off-stoichiometry, alloying on the M site, and interleaving different A elements produce large family diversity (including i-MAX and o-MAX variants). Structural tenability is key to tailoring properties [4]. The chemical bonding of the M-X bond is dominated by M-d/X-p hybridization and is relatively short and directional, being responsible for stiffness, high elastic moduli, and ceramic-like aspects. In contrast, the M-A bond is more metallic and delocalized; it provides ductility and electrical/thermal conductivity and causes metallic behaviors in many MAXs [5]. In terms of electronic properties, metallic behavior predominates for most experimentally realized MAX phases. The Fermi level is typically dominated by M-d states, often hybridized with X or A states. This yields good electrical and thermal conductivity (often anisotropic because conduction along in-plane M-X layers differ from that in the across-layer direction) [5]. The density of states (DOSs) features a pronounced M-d contribution at *E_F_*; the strength of M-X vs. M-A hybridization shifts band filling and can push some phases toward reduced density at *E_F_* (low-DOS metals) or toward instabilities in some compositions [5]. Some compositions (theoretically predicted or engineered via alloying/strain/ordered variants) can have reduced metallicity or pseudo-gap features; by chemical substitution (e.g., heavier A atoms or particular M elements), the electronic character can be modified substantially. In addition, converting MAX into 2D MXene sheets (by selectively etching A) creates a very different electronic landscape (from metals to half-metals or semiconductors, depending on termination) [6]. In recent years, MAX phases have attracted considerable attention as the parent compounds of two-dimensional MXenes, which are obtained by selectively etching the A layers to form M_n+1_X_n_ sheets with various surface terminations such as –O, –OH, and –F [7,8,9,10,11]. This dimensional reduction from layered 3D ceramic–metal hybrids to true 2D materials leads to profound changes in bonding and electronic structure. Most MAX phases are non-magnetic or weakly paramagnetic in experiments because early M elements (Ti, V, etc.) do not provide strong local moments in the MAX lattice [12]. However, notable exceptions arise in Cr- and Mn-based systems, where the partially filled 3*d* orbitals can host robust local magnetic moments. In favorable cases, these moments remain stable, or are even enhanced, upon dimensional reduction to 2D MXenes, enabling ferro- or antiferromagnetic ground states. Several Cr- and Mn-containing MAX phases (e.g., Cr_2_AlC and Mn_2_GaC) have already been synthesized and experimentally confirmed to exhibit magnetic ordering [13,14,15]. Recent theoretical and experimental studies have demonstrated that Cr-containing MXenes exhibit strong spin polarization and correlation-driven magnetic behavior, highlighting the critical role of electron correlation effects across the 3D–2D transition [15,16]. Therefore, a detailed understanding of the structural, electronic, and magnetic properties of Cr-based MAX phases is essential not only for their intrinsic applications, but also for guiding the design of functional magnetic MXenes for spintronic and sensing technologies, and for successful synthesis [3].

Motivated by these considerations, we investigated the electronic structure and a wide range of properties, including structural, magnetic, anisotropic, mechanical, dynamical, and thermal characteristics, of the newly proposed magnetic MAX phase Cr_2_ZnC carbides. Our goal is to stimulate experimental efforts toward the synthesis of these novel compounds, which display promising features for applications such as thermal barrier coatings and refractory materials. Moreover, when Cr_2_ZnC carbides are selectively etched to yield MXene-like Cr_2_C layers with engineered magnetic ordering, they present exciting potential for advanced functional applications, particularly in sensing and spintronic devices.

Before exploring the calculation techniques, it is important to recognize that assessing a material’s stability sometimes requires taking into account competing phases, alternative structures, or configurations that the material can assume under certain conditions. The presence of these competing phases can greatly impact the overall stability and properties of the material. Typically, we identify the most stable competing phases from sources such as the Materials Project [17] and the Open Quantum Materials Database (OQMD) [18]. The analysis revealed that Cr_2_ZnC lies 0.056 eV/atom above the convex hull, with a formation enthalpy of −0.079 eV/atom, and is predicted to decompose into Cr_3_C_2_, with a formation enthalpy of −0.104 eV/atom; Cr_7_C_3_, with a formation enthalpy of −0.106 eV/atom; and Zn, with a formation enthalpy of 0 eV/atom. Although this phase is metastable at 0 K, its hull distance falls well within the commonly accepted metastability range (≤0.3 eV/atom) observed for many experimentally synthesized MAX phases [19]. These findings enhance the robustness of our theoretical predictions by quantitatively establishing their relative thermodynamic stability with respect to competing phases.

However, thermodynamic viability alone is insufficient to guarantee accurate prediction of intrinsic properties, particularly for Cr-based compounds, where strong electron correlations play a decisive role. Transition metal compounds containing partially filled *d* orbitals, such as chromium-based phases, often exhibit strong electron correlations that cannot be adequately described by conventional density functional theory (DFT) within the local density approximation (LDA) or the generalized gradient approximation (GGA). These methods tend to over-delocalize electrons, leading to inaccurate predictions of electronic structures, magnetic ground states, and even erroneous metallic behavior in systems that are experimentally insulating or magnetic. To address this limitation, the DFT+U approach, which incorporates an on-site Coulomb repulsion term (*U*) for localized *d* or *f* electrons, has proven effective in improving the description of magnetic and electronic properties in Cr-based layered materials. For instance, studies on Cr_2_InN, Cr_2_AlC, Cr_2_GaC, and Cr_2_GeC have demonstrated that the inclusion of a Hubbard *U* parameter significantly influences magnetic ordering and enhances the accuracy of structural and electronic predictions [20,21,22]. Given these precedents, it is reasonable to employ both DFT and DFT+U methodologies in the present study of Cr_2_ZnC to rigorously assess the role of electron correlations in determining its structural, electronic, magnetic, and dynamical properties.

## 2. Computational Details

The computational analysis of Cr_2_ZnC and Cr_2_C 2D were carried out using the Cambridge Serial Total Energy Package (CASTEP) [23] code within the framework of density functional theory (DFT) [24]. To accurately describe the electron–ion interactions, OTFG ultrasoft pseudopotentials were employed, with the Perde–-Burke–Ernzerhof (PBE) functional under the generalized gradient approximation (GGA) [25] used to treat exchange–correlation effects, chosen for its proven balance of accuracy and computational efficiency in MAX phase systems. The pseudoatomic electronic configurations considered were Cr (3d^5^4s_1_), Zn (3d^10^4s^2^), and C (2s^2^2p^2^), where applicable. Structural optimization was performed using the limited-memory Broyden–Fletcher–Goldfarb–Shanno (LBFGS) algorithm [26] to minimize the total energy and obtain stable geometries, with convergence ensured via a plane-wave cutoff energy of 650 eV and a 15 × 15 × 3 k-point mesh for Brillouin zone sampling. To account for strong on-site Coulomb correlations in the localized Cr 3d states, we employed the DFT+U method within the rotationally invariant formalism of Dudarev et al. [27], where the correction depends solely on the effective parameter U_eff_ = U − J, with J implicitly set to zero. The Hubbard correction was applied exclusively to the Cr 3d orbitals. For Cr_2_ZnC, a hypothetical compound lacking experimental U or magnetic data, we initially adopted U_eff_ = 2.5 eV, consistent with CASTEP’s default recommendation for Cr and prior studies on Cr-based systems. To assess the sensitivity of our predictions to electronic correlation strength, we performed a systematic U-dependence study over the range Ueff = 0.0–4.0 eV. This approach is physically justified: while U varies significantly with oxidation state and local bonding, J remains relatively insensitive to 3d electron count, supporting the Dudarev treatment. Rather than fitting to unavailable experimental data, this U-scan serves to identify a physically reasonable range that yields stable, self-consistent solutions for structural, magnetic (local moments and ordering), and electronic properties, including the density of states, and aligns with established behavior in Cr-based materials. Based on this analysis, all subsequent calculations for Cr_2_ZnC and its MXene derivative Cr_2_C were carried out using DFT+U with Ueff = 2.5 eV. For geometric optimization, convergence tolerances, maximum stress, maximum force on atoms, and ionic displacement were set to 5 × 10^−7^ eV/atom, 0.02 GPa, 0.01 eV/Å, and 5 × 10^−4^ Å, respectively.

## 3. Results and Discussion

### 3.1. Stability of Cr_2_ZnC (Bulk MAX Phase)

#### 3.1.1. Structural Properties and Stability

**Figure 1:**
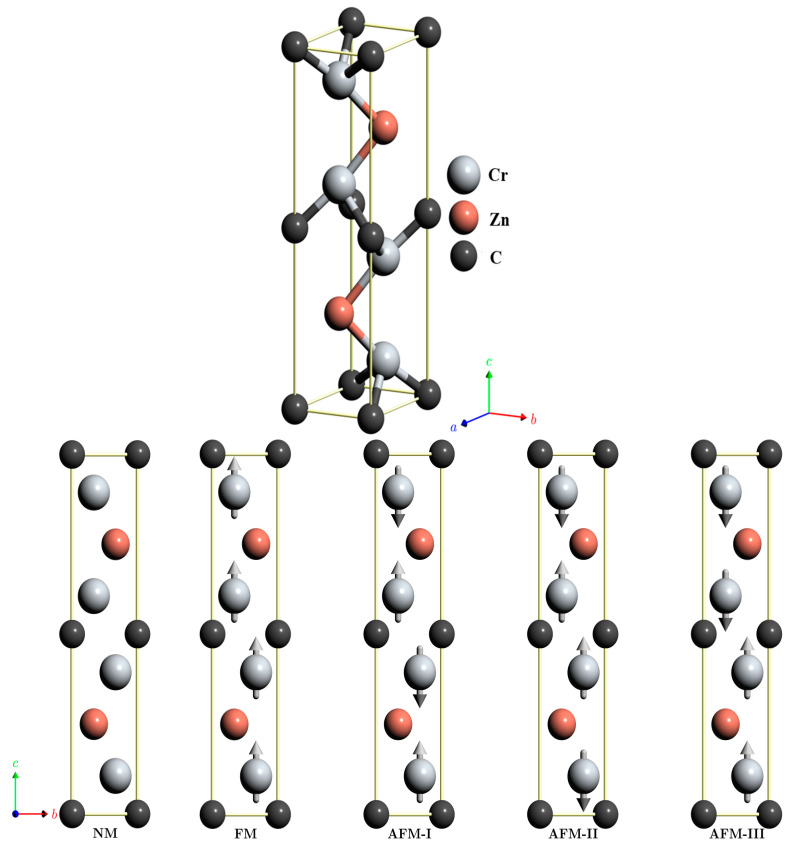
Crystal structure of Cr_2_ZnC.

The hexagonal structure with P6_3_/mmc (no. 194) space group symmetry, as well as the different magnetic orderings (non-magnetic (NM), ferromagnetic (FM), and antiferromagnetic (AFM-I, AFM-II, AFM-III)) of the Cr_2_ZnC MAX phase, are shown in Figure 1.

Each unit cell has eight atoms, and the atomic positions of the Cr, Zn, and C atoms are 4f (2∕3, 1∕3, 0.58), 2d (2∕3, 1∕3, 1∕4), and 2a (0, 0, 1∕2), respectively.

These Cr_2_AlC-type materials consist of layers of closely packed atoms that alternate with layers containing zinc (Zn) and/or transition metal (Cr) atoms.

Before exploring the properties of the magnetic compound under investigation, we first establish its magnetic ground state by comparing the total energies of five configurations—non-magnetic (NM), ferromagnetic (FM), and three antiferromagnetic orderings (AFM-I, AFM-II, AFM-III), under both standard DFT (GGA) and DFT+U. Within standard DFT, the total energy differences between FM and AFM configurations are relatively small (on the order of a few tenths of an eV), indicating weak magnetic competition among several magnetic states. The FM configuration is marginally favored, but AFM-I, AFM-II, and AFM-III remain nearly degenerate, reflecting the itinerant nature of Cr 3d electrons in Cr_2_AC systems. When the on-site Coulomb interaction is included (DFT+U), the magnetic landscape changes significantly. The FM state becomes strongly stabilized and is clearly separated energetically from all AFM and NM configurations. Moreover, the magnetic moment in the FM phase increases dramatically compared to standard DFT, highlighting the enhanced localization of Cr 3d states induced by the Hubbard U correction. In contrast, the AFM configurations exhibit nearly vanishing net magnetic moments, confirming robust antiparallel spin alignment and further emphasizing the sensitivity of Cr-based MAX phases to electron correlation effects. This behavior is consistent with previous reports on Cr_2_AC MAX phases, where standard DFT often underestimates magnetic ordering tendencies, while DFT+U predicts a more stable magnetic ground state with larger local moments. Therefore, the present results confirm that electron correlation plays a decisive role in determining the magnetic ground state of Cr_2_ZnC, and that DFT+U provides a more realistic description of its magnetic properties. Given this strong U-dependence of magnetism, it is essential to assess whether, and how, electron correlation also influences structural stability and energetics, as lattice distortions and formation tendencies may be magnetically driven.

Table 1 presents the calculated structural parameters (lattice constants *a* and *c*, axial ratio *c/a*, and unit cell volume *V*) along with thermodynamic quantities, formation energy (Δ*H*_f_), and cohesive energy (*E*c), for Cr_2_ZnC and selected reference MAX phases (Zr_2_CuB, W_2_CuB, Fe_2_CuB). The results are computed using both the standard GGA and the correlation-corrected GGA+U approach. The formation enthalpies Δ*H_f_* and cohesive energies *E_c_* were computed to determine the thermodynamic stability and synthesizability of compounds using the following formulas [28]:

(1) $${∆H}_{f}\left({Cr}_{2}ZnC\right)=\frac{1}{8}(E_{{Cr}_{2}ZnC}^{tot}-4E_{Cr}^{bulk}-2E_{Zn}^{bulk}-2E_{C}^{bulk})$$

(2) $$E_{C}\left({Cr}_{2}ZnC\right)=\frac{1}{8}(E_{{Cr}_{2}ZnC}^{tot}-4E_{Cr}^{iso}-2E_{Zn}^{iso}-2E_{C}^{iso})$$

where $E^{tot}$, $E^{bulk},$ and $E^{iso}$ are the total energy of the ternary MAX phase carbides Cr_2_ZnC, the total energy per atom in the solid state, and the energy per atom in the isolated state, respectively. Cr_2_ZnC, in both DFT and DFT+U approaches, is thermodynamically stable owing to the negative values of ${∆H}_{f}$ and $E_{c}$, indicating that they can be synthesized.

For Cr_2_ZnC, the two computational methods yield markedly different predictions:

The first one, GGA, predicts a relatively compact structure with *a* = 2.846 Å, *c* = 12.773 Å, and *V* = 89.576 Å^3^. The formation energy is negative (−1.22 eV/atom), suggesting marginal thermodynamic stability, while the cohesive energy (−6.22 eV/atom) indicates moderate bonding strength. In contrast, the second one, GGA+U, significantly expands the lattice, increasing *a* to 3.01 Å and *c* to 13.493 Å, resulting in a larger unit cell volume (105.856 Å^3^). More importantly, it yields a substantially more negative formation energy (−2.68 eV/atom) and comparable cohesive energy (−5.14 eV), confirming that electron correlation effects are essential for accurately describing the structural and energetic stability of this Cr-based compound. This aligns with our earlier findings from band structure and phonon analyses, which also revealed dramatic differences between GGA and GGA+U, particularly in the magnetic moment and electronic structure [31].

According to the computed results, Cr_2_ZnC demonstrates pronounced thermodynamic stability and robust magnetism once electron correlation is incorporated through the GGA+U method, which predicts a strongly negative formation energy (−2.68 eV/atom) and a substantial magnetic moment (12.80 μ_B_). In contrast, the conventional GGA approach yields only a moderately negative formation energy (−1.22 eV/atom) and a much smaller magnetic moment (1.88 μ_B_). In comparison, the M_2_CuB-type MAX phases—namely Zr_2_CuB, W_2_CuB, and Fe_2_CuB—show modest formation energies (ranging from −0.16 to −0.30 eV/atom) and are generally non-magnetic or weakly magnetic, with structural properties dominated by ionic/covalent bonding rather than strong electron correlations. While W_2_CuB displays a high cohesive energy (−9.19 eV/atom) due to tungsten’s robust metallic bonding, it lacks the pronounced spin polarization that makes Cr_2_ZnC uniquely suited for spintronic applications. Thus, Cr_2_ZnC stands apart as a strongly correlated, highly stable, and intrinsically magnetic MAX phase, whose accurate description necessitates beyond-GGA methods, unlike the more conventional M_2_CuB systems.

##### Electronic Structure

Band structure

**Figure 2:**
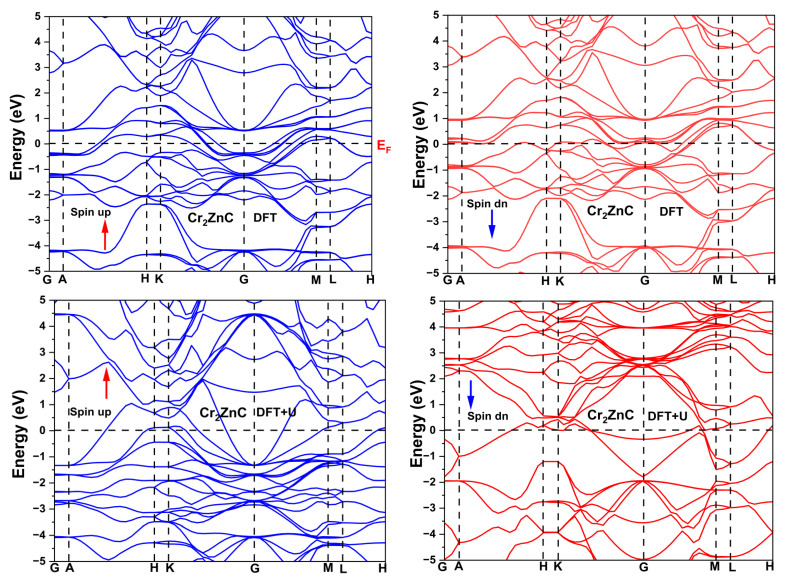
Band structure of Cr_2_ZnC using DFT and DFT+U approaches.

Figure 2 displays the spin-polarized electronic band structures of Cr_2_ZnC obtained within the PBE and PBE+U schemes along the conventional high-symmetry directions of the hexagonal Brillouin zone. In both cases, the material retains a metallic character, as several bands intersect the Fermi level without any indication of a band gap. The dispersion shows that most valence states are mainly located around −3 eV, whereas the states at and near *E_F_* originate mainly from Cr-3d orbitals, with only minor participation from Zn-3d and C-2p states. This electronic distribution reflects strong Cr-C bonding within the octahedral slabs and weaker, more ionic-like interactions between Zn and the Cr-C layers.

A noticeable anisotropy emerges in the band dispersion: steep, highly dispersive bands appear along G-M, and K-G, implying efficient in-plane carrier transport, while flatter bands along G-A, A-H, H-K, and M-L point to more confined out-of-plane electronic states. Within standard DFT, the electronic bands are nearly spin-degenerate, resulting in a weak magnetic response. In contrast, the inclusion of the Hubbard U correction induces a pronounced exchange splitting between the spin channels, leading to the emergence of a sizable magnetic moment. This comparison clearly demonstrates the role of electronic correlation: while the PBE band structure remains weakly spin-polarized, the PBE+U treatment significantly enhances spin splitting and magnetic ordering. This contrast demonstrates that electron correlation is necessary to stabilize magnetism in Cr_2_ZnC and highlights its role in shaping the electronic and magnetic ground state of this MAX phase.

Density of states

**Figure 3:**
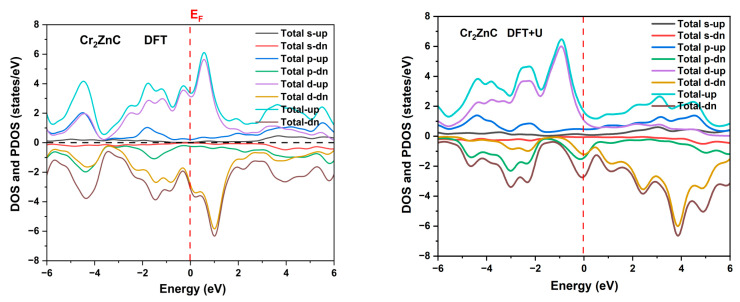
Total density of states (DOSs) and partial PDOS of Cr_2_ZnC using DFT and DFT+U approaches.

The total and partial densities of states (DOSs and PDOSs) of Cr_2_ZnC, computed using both DFT (PBE) and DFT+U approaches (Figure 3), provide critical insight into its electronic structure and magnetic behavior. In both frameworks, the Fermi level (*E_F_*) lies within a region of high DOS, confirming the metallic character deduced from band structure analysis. The dominant contribution near *E_F_* arises from Cr-3d orbitals, particularly in the spin-up channel under DFT+U, which exhibits a pronounced asymmetry between spin-up and spin-down states—consistent with the large magnetic moment (12.80 μ_B_) obtained via the Hubbard correction. In contrast, the DFT-only calculation shows nearly symmetric spin channels and a very small magnetic moment (1.88 μ_B_) compared with DFT+U, underscoring the necessity of including electron correlation effects to capture the true magnetic ground state. The PDOS further reveals that Zn-3d states are localized deep below *E_F_* (~ −8–−10 eV), reflecting their chemically inert, filled-shell nature typical of A-site elements in MAX phases. Meanwhile, C-2p states contribute significantly in the valence region (−5–0 eV), indicating strong covalent bonding between Cr and C atoms. The sharp peaks in the Cr-3d PDOS just below *E_F_* suggest localized electronic states, while broader features above *E_F_* indicate more delocalized conduction states.

Notably, the DFT+U approach introduces a pseudo-gap near −1 eV and enhances the splitting between majority- and minority-spin channels, directly correlating with the stabilization of the magnetic phase. These results not only validate the orbital character assignments made from band structure analysis but also highlight the strongly correlated nature of Cr-3d electrons in Cr_2_ZnC, making it a compelling candidate for applications in spintronics and magnetic devices where precise control over spin-polarized states is essential.

Mulliken Population Analysis (MPA)

As presented in Table 2, the Mulliken population analysis highlights a clear shift in bonding nature and electronic structure when electron correlation is included through GGA+U. Within standard GGA, the C-Cr bond shows a moderate overlap population of 0.94 and a relatively short bond length of 1.947 Å, indicating a partially covalent interaction and weak magnetic polarization on Cr (0.544 μ_B_), with minimal spin contribution from Zn and C. Introducing the Hubbard U increases the C-Cr bond population to 1.04 while simultaneously elongating the bond to 2.082 Å, revealing reduced direct orbital overlap but enhanced charge transfer that strengthens the ionic component of bonding. This in line with the much higher Cr magnetic moment (3.762 μ_B_), which drives the emergence of a large total magnetic moment (12.80 μ_B_) and clear spin splitting in the band structure. Correspondingly, the charge transfer mechanism was further analyzed through Mulliken atomic populations, as shown in Table 3. The results indicates that C becomes more negatively charged (−0.608 e compared to −0.514 e in GGA) with reduced p-character, Zn retains a nearly neutral charge but gains slight spin polarization (−0.489 μ_B_), and Cr d electrons exhibit strong localization. Collectively, these changes demonstrate that GGA+U drives Cr_2_ZnC from a weakly covalent regime toward a more ionic and strongly magnetic bonding configuration, which is crucial for accurately understanding its mechanical, thermal, and magnetic behavior.

#### 3.1.2. Mechanical Stability

##### Elastic Constants and Mechanical Properties

To depict the mechanical characteristics of Cr_2_ZnC compounds, we determined the elastic stiffness constants *C_ij_* and the polycrystalline elastic moduli through the utilization of the strain–stress methodology [32,33] using both DFT (PBE) and DFT+U approaches.

A hexagonal crystal has five stiffness constants: *C*_11_, *C*_12_, *C*_13_, *C*_33_, and *C*_44_, and a sixth dependent one, *C*_66_, which equals (*C*_11_ − *C*_12_)/2. The Born’s stability criteria are given as follows [34]:

*C*_11_ − *C*_12_ > 0, (*C*_11_ + 2*C*_12_)*C*_33_
*>* 2*C*_12_, and *C*_44_ > 0. (3)

The calculated elastic constants of Cr_2_ZnC reveal a significant dependence on the treatment of electron correlation, with the GGA+U approach yielding substantially lower values across all components (*C*_11_, *C*_12_, *C*_13_, *C*_33_, *C*_44_, *C*_66_) compared to standard GGA, indicating that inclusion of the Hubbard U softens the material’s mechanical response. Specifically, C_11_ drops from 375.98 GPa (GGA) to 159.22 GPa (GGA+U), and C_33_ decreases from 108.04 GPa to 35.51 GPa, suggesting reduced resistance to uniaxial compression along the basal plane and c-axis, respectively. This softening is consistent with the expanded lattice volume predicted under GGA+U and reflects the increased ionic/covalent character induced by localized Cr-3d electrons. Despite this reduction, all elastic constants remain positive, satisfying the mechanical stability criteria for hexagonal crystals (*C*_11_ > *|C*_12_*|*, *C*_33_ > 0, *C*_44_ > 0, *C*_66_ > 0), confirming that Cr_2_ZnC remains mechanically stable under both computational schemes. The marked discrepancy between GGA and GGA+U highlights the critical role of electron correlation in accurately modeling the mechanical properties of Cr-based MAX phases, where neglecting U can lead to overestimation of stiffness by nearly a factor of two [35].

To further assess the bonding nature, the Kleinman parameter (*ζ*) was calculated using the following standard equation [36]:

(4) $$\zeta =\frac{C_{11}+8C_{12}}{7C_{11}+2C_{12}}$$

A ζ value close to 0 signifies that bond-bending dominates, while a value near 1 indicates that bond-stretching is more significant.

From Table 4, the Kleinman parameter values, which quantify the relative contribution of bond-bending versus bond-stretching forces in determining a material’s mechanical response, are calculated to be 0.44 under GGA and 0.52 under GGA+U for Cr_2_ZnC. The increase in ζ with the inclusion of the Hubbard U indicates that electron correlation enhances the role of bond-bending deformations relative to bond-stretching, consistent with the softened elastic constants observed under GGA+U. A value of ζ = 0.52 suggests a near-equal balance between these two deformation modes, which may reflect a more flexible or anisotropic bonding environment induced by localized Cr-3d electrons. This shift from ζ < 0.5 (GGA) to ζ = 0.52 (GGA+U) further underscores the sensitivity of Cr_2_ZnC’s mechanical behavior to electronic correlations, making it essential to include U for accurate modeling of its lattice dynamics and mechanical properties.

Table 4 above compares the elastic constants (*Cij*) of Cr_2_ZnC and Cr_2_AlC under both GGA and GGA+U functionals, revealing a pronounced sensitivity to electron correlation in Cr-based systems. For Cr_2_ZnC, inclusion of the Hubbard *U* term drastically reduces *C*_11_ and *C*_33_, underscoring the strong influence of localized Cr 3*d* electrons on the mechanical response; in contrast, Cr_2_AlC exhibits qualitatively similar but quantitatively milder reductions, reflecting comparatively weaker electron correlation in Al-containing systems.

The stiffness constants are further used to calculate the polycrystalline elastic moduli (bulk modulus *B*, shear modulus *G*, and Young’s modulus *E*).

The compressibility modulus *B* and the shear modulus *G* are obtained from the Hill approximation [39], which based on Reuss [34] and Voigt approximations [34], as follows:

*B* = (*B_V_* + *B_R_*)/2,      *G* = (*G_V_* + *G_R_*)/2 (5)

such that:

(6) $$B_{V}=\;\frac{2}{9\;}(C_{11}+C_{12}+2C_{13}+\frac{C_{33}}{2})$$

(7) $$B_{R}=\frac{(C_{11}+C_{12})C_{33}-2C_{12}^{2}}{C_{11}+C_{12}+2C_{33}-4C_{13}}$$

(8) $$G_{V}=\frac{1}{30}\left(C_{11}+C_{12}+2C_{33}-4C_{13}+12C_{44}+12C_{66}\right)$$

(9) $$G_{R}=\frac{15}{4(2S_{11}+S_{33})-4(S_{13}+S_{12})+3(2S_{44}+S_{66})}\;=\frac{\frac{5}{2}\{{\left[\left(c_{11+}c_{12}\right)c_{33}-2c_{12}^{2}\right]}^{2}\}c_{55}c_{66}}{3B_{V}C_{55}C_{66}+[(C_{11}+C_{12})C_{33}-2C_{12}^{2}](C_{55}+C_{66})}$$

where *S_ij_* are the elastic compliance constants.

Young’s moduli *E* and Poisson’s ratios *ν* were predicted using the bulk and shear moduli, as follows [40]:

*E* = 9*B*/(3*B* + *G*),      *ν* = (3*B* − 2*G*)/(6*B* + 2*G*). (10)

The Vickers hardness of the Cr_2_ZnC compound was obtained using the following formula [41]:

(11) $$H_{V}=\;0.92\;{\left(\frac{G}{B}\right)}^{1.137}\times G^{0.708}$$

The mechanical behavior of a material, whether brittle or ductile, can be evaluated using Pugh’s ratio (*B*/*G*). A *B*/*G* value exceeding 1.75 indicates ductility, whereas a lower value suggests brittleness [42]. This observation is further supported by Poisson’s ratio (*ν*), where values above 0.26 are indicative of ductility, while lower values suggest brittleness [42].

Table 5 presents the calculated mechanical moduli (bulk modulus *B*, shear modulus *G*, and Young’s modulus *E*, in GPa), Poisson’s ratio (*ν*), Pugh’s ratio (*B/G*), Vickers hardness (*Hv*, in GPa), and Cauchy pressures(*C*_13_–*C*_44_ and *C*_12_–*C*_66_, in GPa) for the Cr_2_ZnC MAX phase. The mechanical properties of Cr_2_ZnC, as derived from its elastic constants under both GGA and GGA+U approaches, reveal a profound influence of electron correlation on its bulk behavior. While the GGA method predicts a stiff, relatively brittle material with a high bulk modulus (*B* = 182.89 GPa), shear modulus (*G* = 114.82 GPa), and Vickers hardness (*H_V_*) = 15.61 GPa), the inclusion of Hubbard U dramatically softens the material, reducing *B* to 79.49 GPa, G to 47.12 GPa, and *H_V_* to 7.75 GPa, while simultaneously increasing ductility. This is evidenced by the rise in Poisson’s ratio (ν) from 0.240 to 0.25 and Pugh’s ratio (*B/G*) from 1.59 to 1.69, which suggests a transition toward more metallic, ductile character. The Cauchy pressure values further support this trend: under GGA, the negative value of *C*_12_–*C*_66_ (−24.11 GPa) indicates directional bonding and brittleness, whereas GGA+U yields a positive value (11.27 GPa), signaling enhanced plasticity and reduced directional covalency. These results underscore that accurate modeling of Cr_2_ZnC’s mechanical response requires explicit treatment of strong 3d-electron correlations, which significantly alter its stiffness, hardness, and deformation mechanisms compared to standard DFT predictions.

Table 5 compares key mechanical properties of Cr_2_ZnC and Cr_2_AlC under GGA and GGA+U, revealing that electron correlation (DFT +U) significantly softens Cr_2_ZnC: its bulk modulus *B* drops from 182.89 to 79.49 GPa, shear modulus *G* from 114.82 to 47.12 GPa, and Young’s modulus *E* from 284.85 to 118.04 GPa, consistent with Cr’s localized d electrons reducing stiffness. In contrast, Cr_2_AlC shows less dramatic changes under GGA+U, suggesting weaker correlation effects in Al-containing systems.

##### Elastic Anisotropy

To gain deeper insights, the directional dependence of Young’s modulus (*E*), linear compressibility (*β*), and shear modulus has been visually represented in 2D plots. This visualization is achieved using the formula specific to the hexagonal crystal class, as follows [29]:

β = (*S*_11_ + *S*_12_ + *S*_13_) − (*S*_11_ + *S*_12_ − *S*_13_ − *S*_33_)*l*_3_^2^ (12)

1/*E* = (1 − *l*_3_^2^)^2^*S*_11_ + *S*_33_*l*_3_^4^ + *l*_3_^2^(1 − *l*_3_^2^)(2*S*_13_ + *S*_44_) (13)

1/*G* = *S*_44_+ (*S*_11_ − *S*_12_ − 1/2*S*_44_)(1 − *l*_23_) + 2(*S*_11_ + *S*_33_ − 2*S*_13_ − *S*_44_)(*l*_23_ − *l*_43_) (14)

where *S_ij_* represents the compliance matrix and *l*_3_ is the direction cosine, which is given as *l*_3_ = cos *θ* in spherical coordinates.

**Figure 4:**
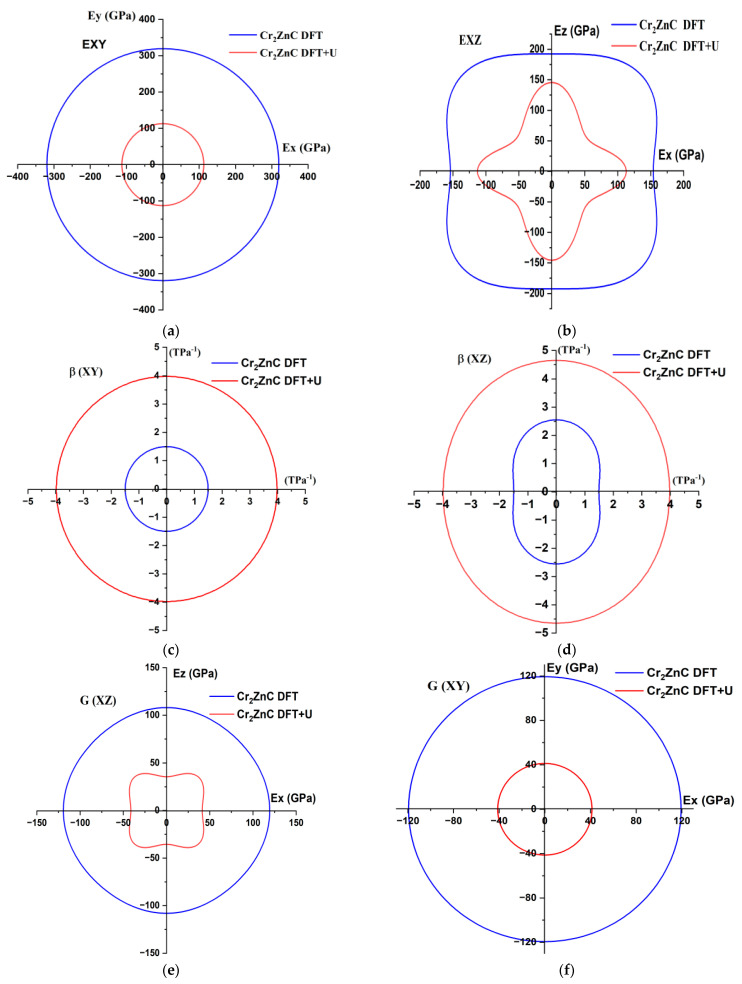
The 2D surface construction of Young’s modulus *E* (**a**,**b**), linear compressibility modulus β (**c**,**d**), and shear modulus G (**e**,**f**) for Cr_2_ZnC using DFT and DFT+U approaches.

The two-dimensional (2D) polar plots of Young’s modulus *E* (a,b), linear compressibility *β* (c,d), and shear modulus *G* (e,f) in the XY and XZ planes (Figure 4) provide a clear and comparative view of the elastic anisotropy of Cr_2_ZnC under both DFT and DFT+U calculations. For Young’s modulus and linear compressibility, the DFT results exhibit nearly circular contours, indicating that the material behaves close to isotropically in these directions. In contrast, the inclusion of the Hubbard U produces noticeably distorted and elongated polar shapes, particularly for β, revealing stronger directional dependence and enhanced anisotropy caused by electronic correlation effects. These distortions reflect the layered nature of Cr_2_ZnC, consistent with elastic constants showing higher in-plane stiffness (*C*_11_ > *C*_33_) and greater compressibility along the c-axis. The shear modulus plots show the opposite trend: while the DFT contours are more irregular and direction-dependent, the DFT+U results become comparatively smoother and more uniform, indicating a reduction in shear anisotropy when correlation effects are included. Altogether, the combined 2D polar representations of E, β, and G highlight how electronic correlations significantly modify the directional mechanical response of Cr_2_ZnC, providing valuable insight for applications where orientation-dependent stiffness, compressibility, and shear resistance are critical.

#### 3.1.3. Dynamical Stability

**Figure 5:**
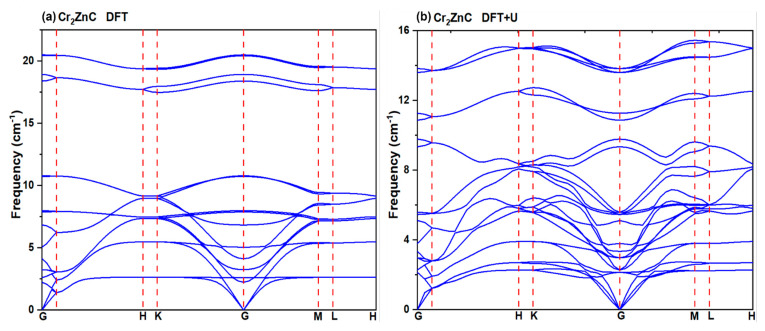
Calculated phonon dispersion spectra of Cr_2_ZnC with (**a**) DFT and (**b**) DFT+U approaches.

Phonon dispersion is an important phenomena of lattice dynamics; the phonon dispersion of a material provide information regarding structural stability and vibrational contribution to thermodynamic properties [44,45]. The dynamical stability of the Cr_2_ZnC compound was further examined through its phonon dispersion spectra calculated using both DFT and DFT+U methods, as shown in Figure 5a,b. The absence of imaginary phonon modes throughout the entire Brillouin zone confirms that Cr_2_ZnC remains dynamically stable under both approaches. The three acoustic branches smoothly converge to 0 THz at the Γ point, while the optical modes extend up to approximately 12.5 THz, reflecting strong lattice rigidity. Below 8 THz, acoustic and low-frequency optical branches show clear coupling without any phonon band gap. Above this range, the optical phonons separate, resulting in a distinct phononic band gap. Atom-projected phonon contributions reveal that the higher optical modes in the 10.85–12.75 THz region are dominated by the lightweight C atoms, the intermediate modes (4–9 THz) originate mainly from Zn vibrations, and the Cr atoms govern both the acoustic region and the lower optical modes (0–4 THz). A comparison of the two computational schemes shows that DFT and DFT+U produce nearly identical phonon dispersions, with only slight upward shifts in high-frequency optical modes when U is applied. This minor stiffening of Cr-C vibrational modes indicates improved treatment of Cr 3d electron localization while maintaining the same qualitative dynamical behavior. Furthermore, several phonon branch crossings near the H, K, and M symmetry points suggest the possible presence of topological phonon features, which deserve further investigation in future work using topologically compatible methods.

### 3.2. Thermal Properties

To characterize a material’s thermal properties, the Debye temperature θ_D_ is commonly used, which may be estimated from the calculated polycrystalline moduli [46].

(15) $$\theta_{D}=\frac{h}{k}{\left[\frac{3n}{4\pi }\left(\frac{N_{A}\rho }{M}\right)\right]}^{\frac{1}{3}}\nu_{m}$$

where ℎ is Planck’s constant, *k* is Boltzmann’s constant, *n* is the number of atoms per molecule, *N_A_* is Avogadro’s number, ρ is the density, M is the molar mass, and ν*_m_* is the average sound velocity. ν*_m_* can be calculated as follows:

(16) $$\nu_{m}={\left[\frac{1}{3}\left(\frac{2}{\nu_{t}^{3}}+\frac{1}{\nu_{l}^{3}}\right)\right]}^{-\frac{1}{3}}$$

where ν_t_ and ν_l_ are the transverse and the longitudinal sound velocity, respectively, obtained from the bulk modulus *B* and the shear modulus *G* as follows:

(17) $$\nu_{t}={\left(\frac{G}{\rho }\right)}^{\frac{1}{2}}\;\;\;\;\;\;\;\;\mathrm{and}\;\;\;\;\;\;\;\;\nu_{l}={\left(\frac{B+\frac{4}{3}G}{\rho }\right)}^{\frac{1}{2}}$$

The calculated Debye temperature for Cr_2_ZnC compounds are listed in Table 6. We can establish the order $\theta_{D}$ as follows: Cr_2_ZnC_GGA_ > Cr_2_ZnC_GGA+U_. According to this order, Cr_2_ZnC_GGA_ has the highest lattice thermal conductivity and the greatest bonding strength [47], whereas Cr_2_ZnC_GGA+U_ shows the lowest thermal conductivity and the weakest chemical bond.

The minimum thermal conductivity *K_min_* is defined as the constant value of thermal conductivity at high temperature. *K_min_* is calculated using Clarke’s model [31]:

(18) $$K_{min}=K_{B}\nu_{m}{\left(V_{atomic}\right)}^{\frac{2}{3}}$$

where *K_B_* is Boltzmann’s constant, *ν_m_* is the average sound velocity, and *V_atomic_* is the cell volume per atom. If *K_min_* > 1.25 W/m K, the material is less likely to have thermal barrier coating applications [48,49].

Table 6 reports key thermal and mechanical parameters of Cr_2_ZnC_GGA_ and Cr_2_ZnC_GGA+U_, including density (ρ), average sound velocity (*v_m_*), Debye temperature (θ_D_), melting temperature (*T_m_*), and minimum thermal conductivity (*K_min_*). Among them, Cr_2_ZnC_GGA_ exhibits the highest Debye temperature (607.14 K) and sound velocity (4557.03 m/s), suggesting stronger atomic bonding and higher phonon frequencies. In contrast, Cr_2_ZnC_GGA+U_ shows the lowest Debye temperature (399.11 K), indicating weaker interatomic interactions and enhanced phonon scattering, leading to reduced thermal conductivity. Cr_2_ZnC_GGA_ has the highest density (6.725 g/cm^3^) and melting temperature (1880.52 K), suggesting greater structural stability, yet its *K_min_* (0.689 W/mK) is higher than that of Cr_2_ZnC_GGA+U_ (0.429 W/mK), likely due to differences in phonon scattering mechanisms. Cr_2_ZnC_GGA+U_, with the lowest density, sound velocity, and Debye temperature, also has the lowest *K_min_* (0.429 W/mK), implying weaker bonding and enhanced phonon scattering. Overall, the results highlight the strong correlation between bonding strength, Debye temperature, and thermal transport properties in these materials.

It can be seen from Table 6 that the calculated minimum thermal conductivities using both approaches Cr_2_ZnC_GGA_ and Cr_2_ZnC_GGA+U_ are less than 1.25 W/m K. As a result, the Cr_2_ZnC MAX phase studied is likely to have thermal barrier coating applications. The melting temperature *T_m_* of a material depends on atomic bonding srtength and the thermal expansion coefficient α. A high *T_m_* denotes strong atomic bonding and a low thermal expansion coefficient α [50]. The *T_m_* can be calculated using the following equation [46]:

(19) $$T_{m}=354+1.5(2C_{11}+C_{33})$$

As shown in Table 6, the melting temperature of Cr_2_ZnC_GGA_ is higher than that of Cr_2_ZnC_GGA+U_ due to strong atomic bonding.

Finally, the lattice thermal conductivity *K_ph_* is one of the most important indicators used to describe the thermal behavior of materials. *K_ph_* can be calculated according to Slack’s model [51] with the following empirical formula:

(20) $$K_{ph}=A\frac{M_{av}\delta \theta_{D}^{3}}{\gamma^{2}Tn^{\frac{2}{3}}}$$

where *M_av_* refers to the average atomic mass of the atom (in kilograms per mole), δ is the volume per atom, $\theta_{D}\;$is the Debye temperature, *T* denotes the absolute temperature in *K*, *n* is the number of atoms per unit cell, γ is the Grüneisen parameter calculated using Poison’s ratio (ν), and *A* is a component associated with γ. γ and *A* can be calculated as follows [52]:

(21) $$\gamma =\frac{3\left(1+\nu \right)}{2\left(2-3\nu \right)}$$

(22) $$A=\frac{2.43\times 10^{8}}{1-\frac{0.514}{\gamma }+\frac{0.228}{\gamma^{2}}}$$

**Figure 6:**
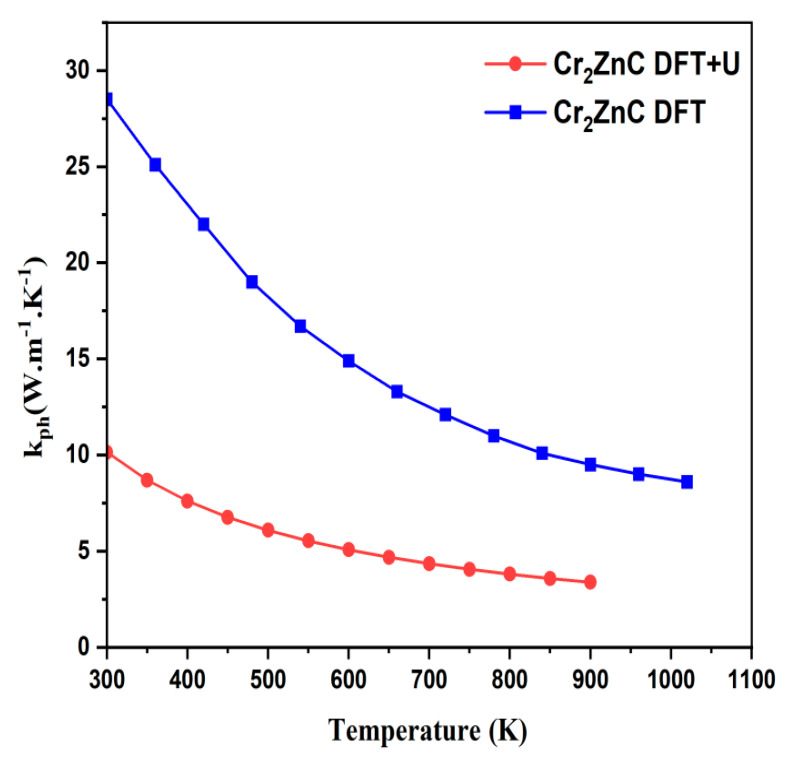
Temperature dependence of calculated lattice thermal conductivity. *K_ph_* for Cr_2_ZnC using DFT and DFT+U approaches.

The lattice thermal conductivities of Cr_2_ZnC phases calculated using both approaches are plotted as a function of temperature (1020 K and 900 K) and are shown in Figure 6. The lattice thermal conductivity *K_ph_* of these compounds follows a general decreasing trend with increasing temperature, which is expected due to intensified phonon–phonon scattering at higher thermal energies. Cr_2_ZnC_GGA_, exhibits the highest *Kph* across all temperature ranges, reaching 26 W/mK at room temperature, as major MAX phases ranges between 12 W/m K and 60 W/m K [53], indicating superior heat transport efficiency and lower phonon resistance. Conversely, Cr_2_ZnC_GGA+U_ has the lowest *K_ph_* (10 W/mK at 300 K), indicating increased phonon scattering [54,55] and lower thermal transport efficiency, which is advantageous for applications like thermoelectrics, where retaining heat on one side of the material is beneficial for performance.

### 3.3. Exfoliation of the Cr_2_C Monolayer

**Figure 7:**
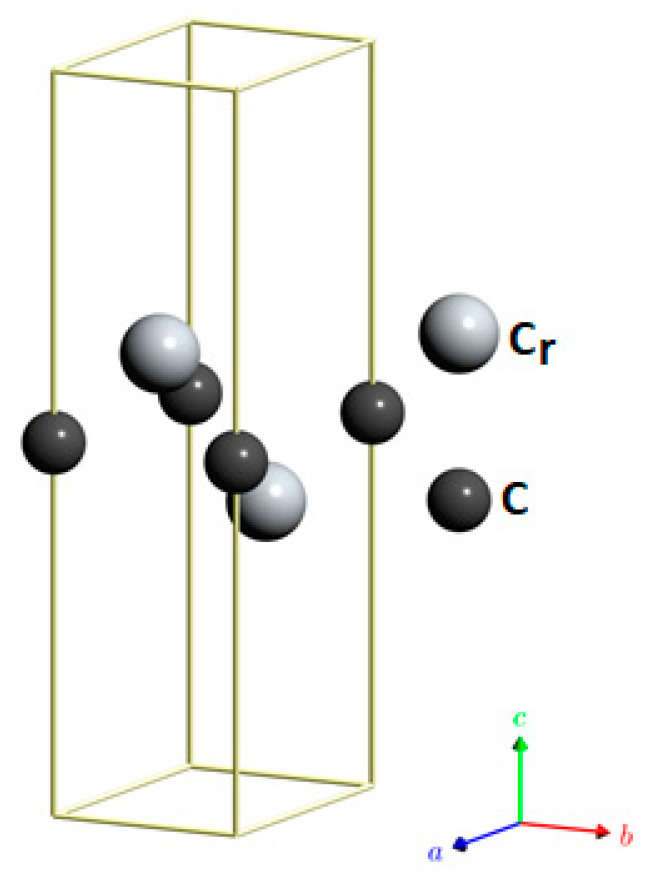
Crystal structure of MXene Cr_2_C.

Two-dimensional (2D) materials continue to attract intense scientific interest due to their transformative potential across diverse fields, including nanoelectronics, thermoelectrics, catalysis, gas storage, ultrasensitive sensing, and advanced functional coatings [56]. This broad applicability has driven extensive efforts to discover and engineer novel 2D systems with tailored properties. Among the strategies employed, the top-down exfoliation of layered precursors remains a prominent route, particularly for materials featuring weak van der Waals forces between layers. Classic examples include the mechanical cleavage or liquid-phase sonication of graphite to yield graphene, as well as similar approaches applied to hexagonal boron nitride and transition metal dichalcogenides [57]. More recently, researchers have extended exfoliation techniques to non-van der Waals systems by leveraging selective chemical etching. Notably, certain MAX phases, layered ternary ceramics known for their exceptional electrical conductivity, mechanical resilience, and strong interlayer bonding, have been successfully delaminated into 2D nanosheets using hydrofluoric acid (HF) or fluoride-based etchants [58]. In this context, the hexagonal MAX phase Cr_2_ZnC (bulk) has emerged as a promising candidate: its A-layer (Zn) can be selectively removed via chemical etching, enabling the isolation of atomically thin Cr_2_C monolayers (Figure 7), a new class of exfoliable 2D transition metal carbides with potential for next-generation applications.

#### 3.3.1. Exfoliation Energy

At present, static calculations are considered as the only way that can provide some useful information on the exfoliation process. In such calculations, the exfoliation energy is defined as follows:

(23) $${∆H}_{f}\left({Cr}_{2}ZnC\right)=\left(\frac{1}{2}E_{{Cr}_{2}ZnC}^{bulk}-E_{{Cr}_{2}C}^{bulk}-E_{Zn}^{bulk}\right)$$

$${∆H}_{f}=-6741.162560845\;-\;(-4952.876327861\;-\;1785.8814)\;=\;-6673.692\;+\;6671.241\;=\;-2.451\mathrm{eV}.$$

where *E_tot_*(*Cr*_2_*ZnC*), *E_tot_*(*Cr*_2_*C*), and *E_tot_*(*Zn*) stand for the total energies of bulk Cr_2_ZnC, 2D Cr_2_C, and the ‘Zn’ element, respectively. The total energy of an ‘Zn’ element is estimated from its most stable bulk structure [59].

#### 3.3.2. Electronic Properties of the Cr_2_C Monolayer

**Figure 8:**
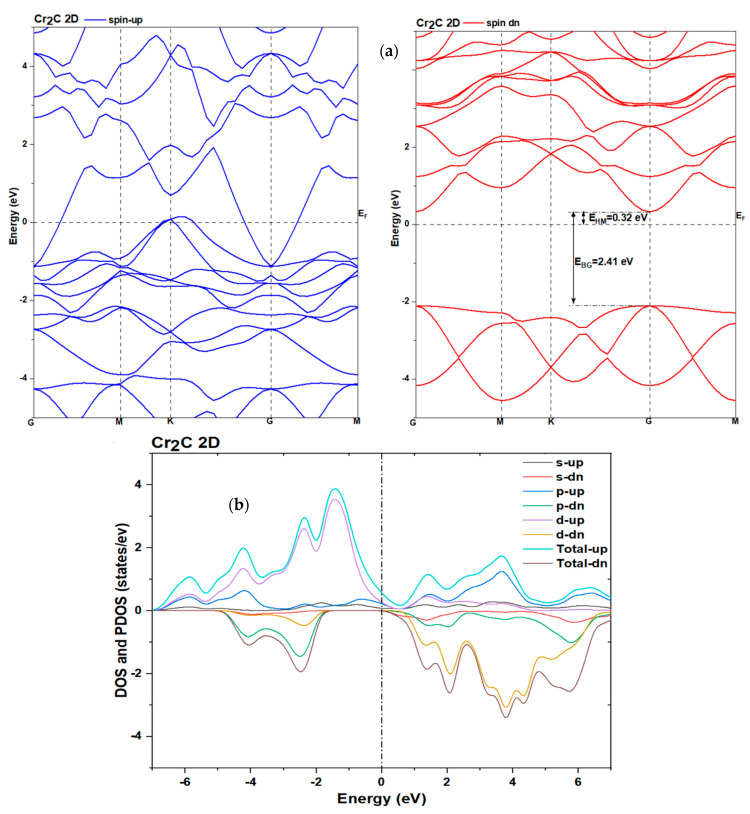
Two-dimensional Cr_2_C using DFT+U approach. (**a**) Structure bands spin-up and spin-down. (**b**) Total density of states (DOSs) and partial PDOS.

To elucidate the electronic origin of the magnetic behavior and conductivity in the Cr_2_C monolayer, we computed its spin-polarized band structure and projected density of states (PDOSs) using DFT+U (with U = 2.5 eV applied to Cr-3d orbitals). The results are shown in Figure 8.

##### Band Structure and Density of State (DOS)

The band structure reveals metallic character in both spin channels, with multiple bands crossing the Fermi level along the high-symmetry path G → M → K → G in the hexagonal Brillouin zone. Notably, the spin-up and spin-down bands are significantly split, confirming strong exchange interactions and consistent with the ferromagnetic ground state.

The spin-polarized band structure of the 2D Cr_2_C monolayer exhibits a clear half-metallic character: the spin-up (majority) channel is metallic, with multiple bands crossing the Fermi level, while the spin-down (minority) channel displays a direct band gap of 2.41 eV at the G point, defined by a well-separated valence band maximum and conduction band minimum, confirming its semiconducting nature. Moreover, in the spin-down channel, the Fermi level is situated near the middle of this gap rather than being shifted toward either band edge, indicating that the electronic response does not favor donor-type (n-type) or acceptor-type (p-type) behavior. Instead, the gap positioning reflects an intrinsic-like semiconductor, in which neither electrons nor holes dominate the carrier population. The total DOS (Figure 8b) shows a pronounced asymmetry between spin channels near *E_F_*, with a higher density of states for spin-up electrons—a hallmark of half-metallic or strongly spin-polarized ferromagnets. The PDOS decomposition indicates that the states near *E_F_* are dominated by Cr-3d orbitals, with minor contributions from C-2p states.

Strong Cr-C hybridization occurs in the energy range from −6 eV to −1 eV, suggesting covalent bonding character. The magnetic moment arises primarily from the unpaired electrons in the Cr-3d spin-up states, in agreement with the atomic-resolved magnetic moments reported in Table 7.

For the DOS (state density) graph, the spin-up channel displays a non-zero DOS at *E_F_*, while the spin-down channel displays a zero (or very low) DOS. Because, for one spin channel (down), there are no states at the Fermi level, electrons of that spin cannot carry the current; it is effectively insulating for that spin. The other spin channel (up) has states *at E_F_*, so metallic conduction occurs only for this spin. Thus, the conduction is dominated by single spin orientation, with a spin polarization close to 100% at *E_F_*. In the context of a device, we can inject almost entirely spin-polarized current (if the surfaces and interfaces behave ideally). The existence of this gap (in the minority channel) ensures that only one spin channel contributes to conduction at *E_F_*. This results in an extremely high-spin bias:

(24) $$P=\frac{N_{↓}\left(E_{F}\right)-N_{↑}\left(E_{F}\right)}{N_{↓}\left(E_{F}\right)+N_{↑}\left(E_{F}\right)}\;$$

for an ideal half-metal. From a spintronics perspective, we can use Cr_2_C 2D as a spin injector or spin filter electrode. $N_{↓}\left(E_{F}\right)=0\Rightarrow P=1$. The magnitude of the half-metallic gap (i.e., the gap in the minority channel at *E_F_*) affects robustness: if the gap is large, thermal excitations, interface states, or defects are less likely to degrade the spin polarization. For Cr_2_C 2D, the gap is large; therefore, we can predict spin polarization as almost ideal.

##### Magnetic Properties of the Cr_2_C Monolayer

The Cr_2_C monolayer exhibits robust ferromagnetic (FM) ordering, as confirmed by spin-polarized DFT+U calculations. The magnetic moment arises predominantly from the Cr 3d orbitals, with negligible contribution from C atoms.

Each Cr atom in Cr_2_C carries a magnetic moment of approximately 4.50 μ_B_, consistent with a high-spin d^4^ electronic configuration, yielding a total magnetic moment of 9.00 μ_B_ per unit cell and indicating robust ferromagnetism. The carbon atoms show small induced spin polarization (−1.01 μ_B_), which likely arises from hybridization with the Cr 3d orbitals. The spin-resolved density of states (Figure 8) demonstrates a pronounced exchange splitting of the Cr 3d bands near the Fermi level, metallic behavior for the spin-up channels, semiconducting behavior for the spin-down channel, and strong d-d hybridization that stabilizes long-range magnetic order. These combined features highlight Cr_2_C as a promising candidate for two-dimensional ferromagnetic materials suitable for spintronic applications requiring room-temperature magnetic stability and efficient electronic transport.

##### U-Dependence

**Figure 9:**
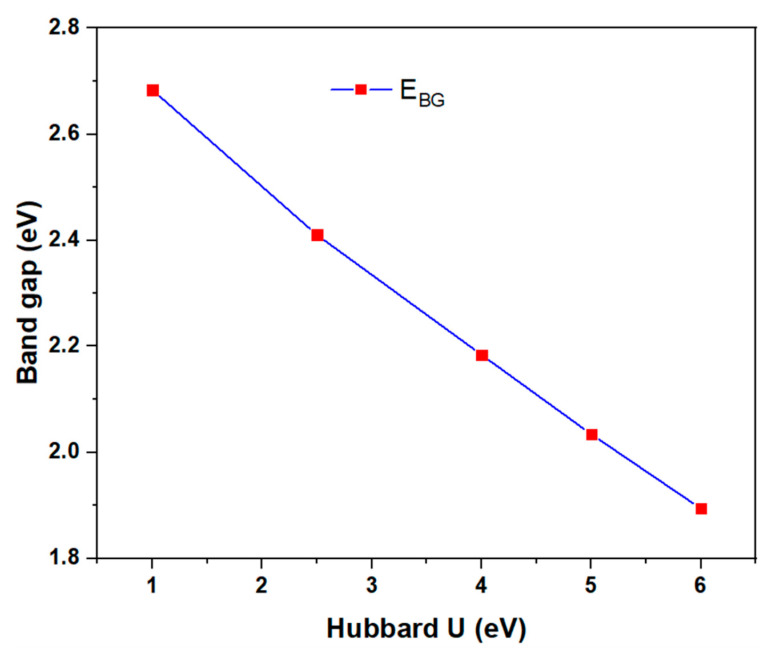
Variation in spin-down band gap of Cr_2_C as a function of the applied Hubbard U.

Variation in the Hubbard U correction does not affect the half-metallic property but influences the width of the spin-down band gap. Increasing U leads to a decrease in the value of the gap band (Figure 9), while the total magnetic moment is essentially independent of U, remaining almost constant at 8.00 μ_B_.

## 4. Conclusions

In this work, we present a comprehensive first-principles investigation of the structural, electronic, magnetic, mechanical, dynamical, and thermal properties of the newly proposed Cr_2_ZnC MAX phase carbide and its two-dimensional MXene derivative, Cr_2_C. Our analysis confirms that bulk Cr_2_ZnC is metallic and thermodynamically viable, with robust mechanical and dynamical stability—evidenced by negative formation energies, the absence of imaginary modes in phonon dispersions, and elastic constants satisfying the Born stability criteria.

Crucially, spin-polarized calculations reveal a dramatic sensitivity to electron correlation effects in this Cr-based system. While the standard PBE functional predicts a small magnetic moment (1.88 μ_B_), inclusion of on-site Coulomb interactions via PBE+U yields a remarkably high magnetic moment of 12.80 μ_B_, highlighting the essential role of strong 3d-electron correlations in accurately describing its magnetic ground state. These correlation effects also significantly influence elastic and thermal properties, underscoring the limitations of conventional DFT for Cr-rich carbides.

Furthermore, our calculations confirm the thermodynamic feasibility of exfoliating Cr_2_ZnC into the 2D MXene Cr_2_C, supported by favorable exfoliation energetics. The resulting monolayer retains robust ferromagnetism, with a large magnetic moment of 8.00 μ_B_. Most significantly, Cr_2_C exhibits intrinsic half-metallicity, as predicted via the GGA+U approach, with a metallic majority-spin channel and a 2.41 eV direct band gap in the minority-spin channel, yielding ~100% spin polarization at the Fermi level.

In direct comparison, while the parent MAX phase Cr_2_ZnC offers high thermodynamic stability and a larger total moment (12.88 μB/f.u.), Cr_2_C combines robust ferromagnetism with enhanced spin polarization at the Fermi level. Its 2D nature enables nanoscale device integration, and its half-metallic character ensures fully spin-polarized currents.

Such half-metallic behavior positions Cr_2_C as a compelling candidate for next-generation spintronic applications. In magnetic tunnel junctions (MTJs), spin valves, and magnetic random-access memory (MRAM), Cr_2_C could serve as a highly efficient spin injector or filter, enabling low-power, non-volatile memory and logic devices beyond conventional charge-based electronics. Its fully spin-polarized current also makes it attractive for spin transistors and spin-based quantum computing architectures, where electron spin states are coherently manipulated for information processing. Beyond spintronics, the spin-selective transport in Cr_2_C holds promise for spin-dependent electrocatalysis and ultrasensitive magnetic sensing. Moreover, the coexistence of metallic and insulating (spin-resolved) behavior within a single 2D material opens avenues for designing multifunctional heterostructures, integrating magnetic, electronic, and optoelectronic functionalities in atomically thin platforms.

## Figures and Tables

**Table 1:** Calculated lattice parameters (*a*) and (*c*) in Å, *c*/*a*, unit cell volume *V* (Å)^3^, formation energy ${∆H}_{f}$ (eV/atom), cohesive energy $E_{c}$ (eV/atom), and magnetic moment (MM) in *µ_B_* from GGA and GGA+U approaches.

Compound	Structural Parameters							
	Approach	a (Å)	c (Å)	c/a	$V{\left(Å\right)}^{3}$	$∆H_{f}$	$E_{c}$	MM
Cr_2_ZnC	GGA	2.846	12.773	4.49	89.576	−1.22	−6.22	1.88
	GGA+U	3.010	13.493	4.48	105.86	−2.68	−5.14	12.80
Cr_2_InN ^a^	GGA(U = 0)	3.065	12.734		103.58			4.11
	GGA+U(U = 1)	3.075	12.77		104.52			4.90
Zr_2_CuB ^b^	GGA	3.493	12.98	3.18	137.15	−0.30	−3.64	0
W_2_CuB ^b^	GGA	3.076	13.02	4.23	106.67	−0.16	−9.19	0
Fe_2_CuB ^c^	GGA	2.784	12.618	4.532	84.664	−0.227		0

^a^ Ref. [20]; ^b^ Ref. [29]; ^c^ Ref. [30].

**Table 2:** Calculated bond overlap populations and bond lengths for Cr2ZnC using GGA and GGa+U approaches.

Compound	Approaches	Bond	Population	Length (Å)
Cr_2_ZnC	GGA	C-Cr	0.94	1.947
	GGA+U	C-Cr	1.04	2.082

**Table 3:** Mulliken atomic populations for Cr_2_ZnC MAX phase carbides using GGA and GGa+U approaches.

Compound		Atom	*s*	*p*	*d*	Total	Charge(e)	Spin (hbar/2)
Cr_2_ZnC	GGA	Cr	Up 1.08	3.37	2.76	7.22	0.110	0.544
			Dn 1.07	3.37	2.23	6.67		
		Zn	Up 0.22	0.63	4.97	5.82	0.293	−0.062
			Dn 0.25	0.67	4.97	5.88		
		C	Up 0.69	1.53	0	2.22	−0.514	−0.082
			Dn 0.70	1.60	0	2.30		
	GGA+U	Cr	Up 1.17	3.44	4.17	8.79	0.183	3.762
			Dn 1.08	3.31	0.63	5.03		
		Zn	Up 0.15	0.50	4.98	5.63	0.242	−0.489
			Dn 0.39	0.76	4.97	6.12		
		C	Up 0.65	1.20	0	1.85	−0.608	−0.901
			Dn 0.78	1.98	0	2.75		

**Table 4:** Calculated elastic constants *C_ij_ (GPa)* and Kleinman parameters *ζ* for Cr_2_ZnC.

Compound	Approaches	*C*_11_	*C*_12_	*C*_13_	*C*_33_	*C*_44_	*C*_66_	*ζ*
Cr_2_ZnC	GGA	375.98	109.25	107.79	265.72	108.04	133.36	0.44
Cr_2_AlC	GGA ^b^ GGA ^a^	373	95	136	375	173		
		396	117	156	382	173		
Cr_2_ZnC	GGA+U	159.22	60.59	27.08	168.81	35.51	49.32	0.52
Cr_2_AlC	(GGA+U) ^b^	340	69	93	472	113		

^a^ Ref. [37], ^b^ Ref. [38].

**Table 5:** Calculated mechanical moduli (GPa), Poisson’s ratio ν, Pugh’s ratio *B/G*, Vickers hardness *H_V_* (GPa), and Cauchy pressures (GPa) for Cr_2_ZnC using two approaches GGA and GGA+U.

Compound								Cauchy Pressure	
	Approaches	*B*	*G*	*E*	*ν*	*B/G*	*Hv*	*C*_13_–*C*_44_	*C*_12_–*C*_66_
**Cr_2_ZnC** **Cr_2_AlC**	GGA ^Exp^ GGA ^a^	182.89 278 191	114.82 116 133	284.85 325	0.24 0.24	1.59	15.61	−0.25	−24.11
**Cr_2_ZnC**	GGA+U	79.49	47.12	118.04	0.25	1.69	7.75	−8.43	11.27
**Cr_2_AlC**	(GGA+U) ^a^	182	131	316					

^a^ Ref. [38], ^Exp^ Ref. [43].

**Table 6:** Density ρ, average sound velocity υm, Debye temperature θ_D_, melting temperature *T_m_*, and minimum thermal conductivity *K_min_* for Cr_2_ZnC.

Compounds	Approaches	*ρ* (g/cm^3^)	*υ_m_* (m/s)	*θ_D_* (K)	*T_m_* (K)	*K_min_* (W/mk)
Cr_2_ZnC	GGA	6.725	4557.03	607.14	1880.52	0.689
	GGA+U	5.691	3173.03	399.11	1084.87	0.429
Cr_2_InC ^a^	GGA+U	7.117	2731.11	340.8	1113.5	0.662

^a^ Ref. [20].

**Table 7:** Mulliken atomic populations for Cr_2_C MAX phases.

Species	Ion	Spin	s	*p*	d	Total	Charge(e)	Spin (hbar/2)
C	1	up:	0.664	0.1.21	0	1.875	−0.757	−1.007
	1	dn:	0.8	2.083	0	2.882		
Cr	1	up:	1.327	3.298	4.437	9.063	0.379	4.504
	1	dn:	1.09	3.134	0.335	4.559		
Cr	2	up:	1.327	3.298	4.437	9.063	0.379	4.504
	2	dn:	1.09	3.134	0.335	4.559		

## Data Availability

The original contributions presented in this study are included in the article. Further inquiries can be directed to the corresponding authors.
